# Case report: management of a young male patient with diabetic ketoacidosis and thyroid storm

**DOI:** 10.3389/fendo.2024.1403893

**Published:** 2024-06-17

**Authors:** Xiaoyu Huang, Yan Chen, Xinwei Huang, Jiahao Tang

**Affiliations:** ^1^ Emergency & Disaster Medicine Center, The Seventh Affiliated Hospital, Sun Yat-sen University, Shenzhen, China; ^2^ Department of Endocrinology, The Seventh Affiliated Hospital, Sun Yat-sen University, Shenzhen, China

**Keywords:** diabetic ketoacidosis, thyroid storm, recurrent diabetic ketoacidosis, autoimmune polyendocrine syndrome, case report

## Abstract

This report describes a case of concomitant diabetic ketoacidosis (DKA) and thyroid storm (TS) in a 20-year-old male patient that presented both diagnostic and management challenges owing to their intricate interrelationship in endocrine-metabolic disorders. The patient, previously diagnosed with type 1 diabetes mellitus (T1DM) and hyperthyroidism, was admitted to the emergency department with symptoms of DKA and progressive exacerbation of TS. Initial treatment focused on correcting DKA; as the disease progressed to TS, it was promptly recognized and treated. This case emphasizes the rarity of simultaneous occurrence of DKA and TS, as well as the challenges in clinical diagnosis posed by the interacting pathophysiological processes and overlapping clinical manifestations of DKA and TS. The patient’s treatment process involved multiple disciplines, and after treatment, the patient’s critical condition of both endocrine metabolic diseases was alleviated, after which he recovered and was eventually discharged from the hospital. This case report aims to emphasize the need for heightened awareness in patients with complex clinical presentations, stress the possibility of concurrent complications, and underscore the importance of prompt and collaborative treatment strategies.

## 1 Introduction

Hyperthyroidism and diabetes mellitus (DM) are both widespread endocrine disorders with profound implications for global public health. Thyroid storm (TS), a crucial and potentially fatal manifestation of hyperthyroidism, is characterized by an abrupt surge in thyroid hormone levels, precipitating severe hypermetabolic symptoms (1). The global prevalence of hyperthyroidism is approximately 0.8%, and while the incidence of TS is relatively low, it is associated with a substantially high mortality rate ranging from 10% to 20% (2). Type 1 diabetes mellitus (DM) is an autoimmune disease characterized by the autoimmune destruction of pancreatic β-cells, necessitating a lifelong dependency on insulin (3). Diabetic ketoacidosis (DKA), a serious complication of DM, manifests with hyperglycemia, elevated ketone bodies, and metabolic acidosis (4).

Several epidemiological studies have revealed a higher prevalence of comorbidity between thyroid disease and diabetes than in the general population, although direct evidence is lacking (5–7). This suggests a complex interrelationship between the pathophysiological processes of hyperthyroidism and diabetes, and the two disorders are often present in the same individual (8). The coexistence of these conditions complicates treatment and increases patient risk, particularly when hyperthyroidism progresses to TS and DM progresses to DKA, requiring rapid recognition and urgent treatment.

Despite the well-established diagnostic approaches and treatment protocols for TS and DKA, limited literature is available on the diagnostic approaches and treatment strategies for their simultaneous presentation. This rare clinical situation poses a significant challenge to clinicians, requiring interdisciplinary knowledge and decision-making skills in the management of acute and critical illnesses. Here, we present a case of concomitant DKA and TS in a young male patient, who recovered and was eventually discharged from the hospital with prompt and effective treatment. The objective of this case report is to share the diagnostic challenges and therapeutic passages when TS and DKA co-occur to inform clinical practice.

## 2 Case presentation

A 20-year-old male patient was admitted to the emergency department 2 months ago with suspected DKA. The patient was diagnosed with T1DM ketoacidosis and hyperthyroidism 4 years ago, following the onset of symptoms such as polydipsia, polyuria, and xerostomia. Unfortunately, specific details regarding the initial diagnostic and treatment process as well as prior medical records are unavailable. He was treated with rehydration and glucose-lowering therapies during the hospital stay. Following discharge, the patient was initiated on subcutaneous insulin therapy. However, he exhibited frequent insulin omissions, approximately 4–5 times per week, leading to inadequate glycemic control. Additionally, the patient was prescribed methimazole treatment for 9 months but discontinued it based on medical advice without subsequent thyroid function tests or related antibody assessments. One year ago, the local hospital adjusted the insulin regimen to insulin aspart 15 units subcutaneously three times daily and insulin detemir 30 units subcutaneously nightly, with no accompanying blood glucose monitoring. He presented to the emergency room primarily because of acute medical concerns—a significant 3-day history of fever, vomiting, and abdominal pain. He reported 8–10 episodes of non-bilious vomiting without hematemesis, associated with generalized abdominal pain. Further, he described the abdominal pain as intermittent, colicky, 7/10 on the numeric rating scale, and non-radiating. He denied having cough, expectoration, sore throat, rhinorrhea, diarrhea, melena, and dysuria. Importantly, he had discontinued his subcutaneous insulin therapy 3 days prior to this admission.

In the emergency room, he was conscious with a temperature of 36.4°C, pulse of 145 beats/min, respirations of 35 breaths/min, blood pressure of 135/69 mmHg, SPO2 of 99% on room air, and a Glasgow Coma Scale (GCS) score of 15. He had generalized dry, warm skin, an enlarged neck, a grade 2 enlargement of the thyroid gland with no palpable nodules, and no vascular murmur. The lungs were clear on auscultation, heart rate was 145 beats/min with regular rhythm, and heart sounds were hyperdynamic. The abdominal muscles were tense, and there was tenderness and rebound pain throughout the abdomen. There was no bilateral lower extremity edema. Initial laboratory data indicated significant metabolic acidosis, with a plasma glucose level of 20.4 mmol/L and β-hydroxybutyrate level of 6.76 mmol/L. Arterial blood gas analysis showed a pH of 7.21, an anion gap of 19 mmol/L, and a delta ratio of 0.65. Thyroid function tests revealed thyrotropin at <0.01 μIU/mL (0.60–4.40), free triiodothyronine at 9.86 pg/mL (2.25–3.58), and free thyroxine at >5.00 ng/dL (0.85–1.38). Additionally, infectious markers such as leukocytes and neutrophil ratio and other biochemical tests were elevated (detailed in **Table 1**). The electrocardiogram displayed marked sinus tachycardia. Abdominal computed tomography scans revealed diffuse severe fatty liver, that did not reveal any findings necessitating surgical intervention. According to the Burch–Wartofsky Point Scale (BWPS) (1) and the Japanese Thyroid Association’s (JTA) guidelines for the management of TS (9), our patient met the diagnostic criteria for TS which included a score of 45 points and TS2, respectively. Because he did not exhibit persistent hyperthermia and had a normal temperature in the emergency room, he was believed to be experiencing inflammation as a result of a bacterial infection. Conversely, his tachycardia and gastrointestinal symptoms were attributed to DKA, leading to a diagnosis of DKA and pre-TS. Consequently, he was transferred to the endocrinology ward for further treatment.

**Table 1:** Summary of patient’s laboratory test results.

Test Name	11/29/2023 Day 1	11/30/2023 Day 2	12/01/2023 Day 3	12/04/2023 Day 6	12/07/2023 Day 9	Normal Reference Range
WBC	14.23	12.03	7.50	5.46	6.57	3.5–9.5×10^9/L
NEUT%	79.9	87.6	71.9	33.5	44.4	40.0–75.0%
Hb	131	126	129	148	142	130–175 g/L
PLT	285	263	178	196	203	125–350×10^9/L
CRP	11.86	25.10	10.79	<10.00	<10.00	< 10.00 mg/L
PCT	0.34	0.35	3.21	0.33	–	< 0.05 ng/mL
BC	Negative	–	–	–	–	negative
PH	7.21	7.38	7.25	7.36	7.34	7.35–7.45
Na^+^	127	134	133	134	138	137–147 mmol/L
K^+^	4.60	3.94	3.92	3.33	4.26	3.50–5.30 mmol/L
Glu	20.40	7.94	10.80	5.88	9.66	3.90–6.10 mmol/L
CO_2_	6.07	19.57	11.57	20.78	23.83	20–30 mmol/L
BHB	6.76	0.49	6.41	0.46	0.25	<0.30 mmol/L
HbA1c	9.9	–	–	–	–	4.0–6.4%
ALT	43.11	–	–	50.00	47.28	9–50 U/L
AST	37.17	–	–	51.74	43.37	15–45 U/L
TBIL	20.29			14.14	16.54	<23.00 µmol/L
TSH	<0.01	–	–	–	–	0.60–4.40 μIU/mL
FT3	9.86	–	–	–	–	2.25–3.58 pg/mL
FT4	>5.00	–	–	–	–	0.85–1.38 ng/dL
TRAb	19.17	–	–	–	–	<1.75 IU/L
TgAb	10.50	–	–	–	–	<4.11 IU/mL
TPOAb	>1000.00	–	–	–	–	<5.61 IU/mL
GADA	positive	–	–	–	–	negative

WBC, white blood cell count; NEUT%, neutrophil percentage; Hb, hemoglobin; PLT, platelet count; CRP, c-reactive protein; PCT, procalcitonin; BC, blood culture; PH, blood pH; Na+, sodium; K+, potassium; Glu, glucose; CO2, carbon dioxide; BHB, β-hydroxybutyric acid; HbA1c, glycated hemoglobin; ALT, alanine aminotransferase; AST, aspartate aminotransferase; TBIL, total bilirubin, TSH, thyroid stimulating hormone; FT3, free triiodothyronine; FT4, free thyroxine; TRAb, thyroid receptor antibodies; TgAb, thyroglobulin antibodies; TPOAb, thyroid peroxidase antibodies; GADA, anti-glutamic acid decarboxylase antibodies.

In the endocrinology ward, he received continuous intravenous infusion of insulin to maintain glycemic control and aggressive intravenous fluid therapy to replenish lost fluids. Additionally, he was administered propylthiouracil 100 mg (t.i.d) to control thyroid function, propranolol 10 mg (t.i.d) to regulate his heart rate, and piperacillin tazobactam as the antibiotic. During treatment, the patient’s heart rate gradually ranged between 113–123 beats/min, and his blood pressure fluctuated between 146 and 156/84–89 mmHg. Concurrently, his body temperature peaked at 37.6°C, which eventually returned to normal without intervention. Approximately 10 hours after admission, the patient experienced a sudden onset of coughing, accompanied by the expulsion of pink, frothy sputum, shortness of breath, agitation, a heart rate of 128 beats/min in sinus rhythm, a respiration rate of 25 beats/min, and a decrease in pulse oximetry to 88%. Furthermore, chest auscultation revealed bilateral wet rales and slight wheezing, and bedside chest radiography was promptly performed, which showed bilateral pulmonary markings and scattered patchy infiltrates around both pulmonary hila, leading to the diagnosis of acute heart failure. Accordingly, he was administered deacetyldigoxin injection, furosemide, and nitroprusside intravenously, and budesonide via nebulization to alleviate airway spasm. Following this treatment, the patient’s cough and shortness of breath improved, and he was immediately transferred to the intensive care unit for further assessment and care.

In the intensive care unit, the patient experienced hyperthermia, with a peak temperature of 39.7°C accompanied by diarrhea, blood pressure of 160/95 mmHg, and a heart rate of >150 beats/min in sinus rhythm. At this time, the patient’s BWPS score reached 85, and the JTA criteria met TS1, which indicated the presence of TS. The patient was administered an intravenous push of dexamethasone injection 2 mg every 6 h, along with oral propylthiouracil tablets at a dosage of 200 mg every 8 h, and an oral compound iodine solution at a dosage of 0.5 mL every 6 h, containing 63.5 mg of iodine. In addition, intravenous esmolol was used to control the ventricular rate, and other appropriate treatments were provided. Post-treatment, the patient experienced relief from abdominal pain and a return to normal body temperature, with the heart rate dropping to a rate of 100–110 beats/min. The patient was subsequently re-transferred to the Department of Endocrinology. Upon transfer, iodine was discontinued, and the dosage of glucocorticoids was gradually reduced until they were completely discontinued. Additionally, the dosage of propylthiouracil was reduced to 100 mg every 8 h. Follow-up assessments revealed thyrotropin receptor antibodies at 19.17 IU/L (normal range: <1.75), anti-glutamic acid decarboxylase antibodies (+), and glycosylated hemoglobin levels of 9.9%. During his hospitalization, ketoacidosis with normal blood glucose levels was observed on several occasions, necessitating the administration of strengthened parenteral nutrition and subcutaneous insulin injections to maintain blood glucose levels between 8 and 12 mmol/L. After the patient was transferred back to the endocrinology ward, we conducted systematic patient education to help him better understand the objectives of various treatment measures and their potential impact on his condition. After 9 days of hospitalization, the patient’s health improved. His blood glucose levels were maintained within the target range of 8–12 mmol/L, multiple re-evaluations of blood gas analyses indicated a return to normal acid-base balance, and the anion gap was consistently within the normal range. Additionally, repeated tests for both blood and urine ketones were negative. Based on these clinical and laboratory findings, we concluded that the patient’s DKA had been effectively resolved. The patient was subsequently discharged from the hospital and instructed to continue receiving subcutaneous insulin injections to lower glucose levels and to switch from propylthiouracil to methimazole for the treatment of hyperthyroidism. After discharge, the patient continued his recovery in a home environment. He reported that with proper medication management over time, there was a significant improvement in his quality of life.

## 3 Discussion

The relationship between DM and thyroid disease is a complex one, involving several genetic, biochemical, and pathophysiological factors. Abnormal thyroid function has been identified as a risk factor for DM, and it can also complicate disease progression and treatment. It is crucial to diagnose and treat diabetic patients who have concomitant thyroid disease as early as possible to prevent further complications and optimize outcomes.

A systematic review has indicated that the yearly incidence of DKA among individuals with T1DM encompasses a spectrum ranging from 0 to 56 instances per 1,000 individuals (10). Furthermore, a study from Japan reported a yearly incidence of TS in hospitalized patients at 0.2 cases per 100,000 individuals, along with a notably high mortality rate of 10.4% (11). Noteworthy catalysts for TS onset include irregular utilization of anti-thyroid medications, infectious etiologies, occurrences of DKA, surgical interventions, radioiodine therapy, adrenal insufficiency, and administration of iodine-containing contrast agents (1). Among these, the cessation of antithyroid medications, infectious episodes, and occurrences of DKA are particularly prevalent and deemed conceivable triggers for TS in the clinical scenario under consideration. The present case manifests a cascade of events wherein an acute infectious episode precipitated DKA, subsequently culminating in TS. Of paramount concern, an empirical report underscored a mortality rate of 15% in cases where TS coexists with DKA (12). Despite the substantial fatality associated with this complex clinical entity, it is noteworthy that the incidence of TS complicating DKA remains infrequent. However, meticulous statistical data pertaining to this specific intersection of pathologies are not yet readily available.

Hyperthyroidism exerts its influence on glucose metabolism by inducing insulin resistance through the upregulation of glucose production via glycolytic and gluconeogenic pathways, while concurrently enhancing intestinal glucose absorption (13). Additionally, thyroid hormones contribute to lower serum insulin levels by augmenting renal excretion (14). This dual impact results in insulin deficiency and resistance, lifting the inhibition on hormone-sensitive lipase, thereby fostering uncontrolled fat breakdown and increased fatty acid oxidation, ultimately elevating ketone production (15). The amalgamation of these metabolic perturbations manifests as hyperglycemia and DKA, a phenomenon that explains the recurrent episodes of normoglycemic DKA observed in the present case, thereby posing challenges for prompt resolution. In severe cases, the confluence of TS and infection may accelerate β-cell destruction (16). During DKA, the body experiences both inflammatory and physiological stress, further predisposing it to the onset of TS. A comprehensive review was conducted in 2019 by Rathish and Karalliyadda (12), wherein the characteristics of the 26 cases featuring the coexistence of TS and DKA were summarized through database retrieval. While most cases had pre-existing diabetes diagnosis preceding the occurrence of TS, the temporal sequence of DKA antedating TS remains ambiguous. Some scholars posit that excess thyroid hormones precipitate DKA, subsequently culminating in TS (17). Notably, in patients with uncontrolled diabetes, reductions in T3 levels may be attributed to impaired T4-T3 peripheral conversion, a situation potentially alleviated with improvements in blood glucose control (18). Similar observations were made in our patient, marked by significantly elevated T4 levels, mildly elevated T3 levels, and noticeable decrease in TSH levels. The intricate interplay between hyperthyroidism and DKA constitutes a multifactorial process that requires comprehensive medical management and treatment. Specifically in diabetic patients with concomitant thyroid disorders, vigilant monitoring of metabolic status is imperative for timely intervention and prevention of complications.

Grave’s disease (GD) and T1DM share autoimmune underpinnings, constituting prevalent endocrine disorders that are frequently encountered in clinical practice. Remarkably, these conditions often coexist within the same individual. However, the relationship between GD and type 2 diabetes mellitus (T2DM) remains unclear. From a diagnostic perspective, the current case aligns with the criteria characteristic of autoimmune polyendocrine syndrome type 2 (APS-2), necessitating the presence of a minimum of two among the following autoimmune conditions: autoimmune thyroid disease, T1DM, and Addison’s disease (19). Genetic scrutiny of APS-2 has brought to light common genes and single-nucleotide polymorphisms associated with various organ-specific autoimmune diseases. Notably, individuals at risk for celiac disease within the APS-2 spectrum often exhibit variations in *DR3-DQ2* and *DR4-DQ8* (20), haplotypes synonymous with T1DM (21), autoimmune thyroid disease (22), and Addison’s disease (23). Additionally, genes encoding cytotoxic T-lymphocyte-associated protein 4 (*CTLA-4*) (24), protein tyrosine phosphatase (*PTPN1*), non-receptor type 22 (*PTPN22*), transcription regulator protein *BACH2* (25, 26), and CD25-interleukin-2 receptor (27) have been recognized for their association with APS-2 risk. Despite substantial progress in elucidating disease-related genetic factors, the heritability of APS-2 remains complex. A specific method for APS-2 detection is currently unavailable. However, the assessment of autoimmune antibodies has emerged as a valuable tool for evaluating disease risk, because these antibodies are often detectable several years before the onset of clinical manifestations. Among these antibodies, the thyroid peroxidase, glutamic acid decarboxylase, and 21-hydroxylase antibodies are the most prominent.

The incidence of DKA combined with TS is extremely low, and there are no clear criteria for the diagnosis and treatment of the intersection of the two endocrine emergencies. The diagnosis of TS relies on severe thyrotoxic symptoms, including persistent elevated body temperature that is difficult to reduce, tachycardia, congestive heart failure, central nervous system involvement, and gastrointestinal symptoms, among others. Interestingly, in this case, the patient did not have a high temperature in the emergency room and subsequently presented with hyperthermia in the intensive care unit after adequate rehydration. Previous literature reports suggest that this patient was not the only one to develop such a condition (28, 29), which may be related to the occurrence of DKA leading to a decrease in body temperature (30). The mechanism by which DKA causes hypothermia is not yet fully understood, and some studies claim that it is related to the lack of glucose endocytosis due to a deficiency of insulin, which leads to a lack of cellular heat-producing substrate (31). Other studies suggest that patients with DKA have ketones in the body, and the acidosis caused by the bodily accumulation of ketone bodies and lactic acid has an inhibitory effect on the thermoregulatory center (32). Diabetic patients also have impaired thermal insulation, and when combined with autonomic impairment, reflex vasoconstriction is diminished, and they become prone to excessive heat dissipation (33, 34). Perhaps these factors contributed to the patient not showing persistent hyperthermia at the beginning of treatment but only after subsequent DKA control.

Since the initial report of a diabetic coma combined with TS in 1951 (29), numerous similar cases have been documented over the past 70 years. Most of these cases were identified as TS during the patient’s initial clinic visit. However, there are few cases like the one at hand, which were diagnosed as pre-TS during the emergency room visit and subsequently progressed to a TS during hospitalization. It is necessary to emphasize, that the patient attained a BWPS score of 45 in the emergency department and met the TS2 criteria in the JTA owing to his tachycardia and gastrointestinal symptoms being considered associated with DKA, which is a common clinical occurrence in current times. The diagnosis of TS is challenging because of the absence of a specific indicator. This is demonstrated in the case report, where the patient did not exhibit the typical symptoms of TS, such as persistent hyperthermia. It is essential to consider that the effect of DKA on body temperature might have contributed to the patient’s condition. Therefore, we must acknowledge that there is a possibility that the score of TS has been underestimated.

When DKA and TS co-occur, the overlapping symptoms complicate the diagnosis of TS, as both conditions can present with tachycardia, fever, and gastrointestinal symptoms. The complexity of these combined symptoms underscores the absence of established guidelines that delineate clear criteria for distinguishing between active intervention and mere monitoring in such patients. This diagnostic ambiguity necessitates a judicious approach for the management of these patients.

Given the challenges associated with routine screening for thyroid dysfunction in all DKA patients that could lead to increased healthcare costs and resource utilization without commensurate benefits across all clinical settings, a more targeted approach is advisable. Screening should be particularly considered for patients who exhibit specific clinical indicators suggestive of thyroid dysfunction, or when the presentation of DKA is atypical. For instance, subtle signs of TS such as unexplained tachycardia, disproportionate fever, or gastrointestinal symptoms relative to the severity of DKA should trigger a further evaluation for TS.

Therefore, in cases where DKA and suspected TS coincide, it is critical to consider whether to refine existing diagnostic criteria or to adopt a more aggressive intervention approach. This strategy not only ensures efficient resource utilization but also enhances the precision of medical responses, thereby improving patient outcomes. Exercising clinical caution and maintaining vigilance are paramount in these complex clinical scenarios.

## 4 Conclusion

The coexistence of DKA and TS presents a unique clinical challenge that calls for careful clinical judgment and prompt treatment. This case underscores the importance of considering multiple diagnoses in patients with overlapping symptoms of different endocrine emergencies. The atypical presentation of the patient, in which DKA obscured the typical features of TS, highlights the need for clinicians to be highly vigilant regarding multiple concurrent endocrine disease, especially in patients with pre-existing endocrine disease. The management of this patient required coordination among multiple disciplines, including aggressive treatment for both DKA and TS, as well as supportive measures for associated complications. This case adds to the limited literature on the concurrent management of these conditions and emphasizes the importance of timely diagnosis and early intervention in the setting of overlapping endocrine emergencies. Future research should concentrate on developing clear diagnostic and therapeutic guidelines for patients with both DKA and TS to enhance prognosis in this high-risk population.

## Data availability statement

The raw data supporting the conclusions of this article will be made available by the authors, without undue reservation.

## Ethics statement

Written informed consent was obtained from the participant/patient(s) for the publication of this case report.

## Author contributions

XyH: Writing – original draft, Investigation, Conceptualization. YC: Writing – review & editing, Resources. XwH: Writing – review & editing. JT: Writing – review & editing, Supervision.
